# The Predictors of State and Trait Anxiety Among Healthcare Workers During the Coronavirus Disease 2019 Pandemic in Iran: A Cross‐Sectional Study

**DOI:** 10.1002/hsr2.71023

**Published:** 2025-07-09

**Authors:** Azam Sharifi, Masoud Fallahi‐Khoshknab, Shamaneh Mohammadi, Mashaallah Zeraati Nasrabadi, Zahra Jamshidi, Mohsen Aghabeygi‐ Arani, Nilofar Mirzaei, Negin Fallahi‐Khoshknab, Parisa Rasouli, Abbas Ebadi

**Affiliations:** ^1^ Department of Emergency and Critical Care Nursing, School of Nursing and Midwifery Kermanshah University of Medical Sciences Kermanshah Iran; ^2^ Nursing Department University of Social Welfare and Rehabilitation Sciences Tehran Iran; ^3^ Department of Medical Surgical and Geriatric Nursing Kashan University of Medical Sciences Kashan Iran; ^4^ Nursing Department, School of Nursing and Midwifery Shiraz University of Medical Sciences Shiraz Iran; ^5^ Nursing Department, School of Nursing and Midwifery Lorestan University of Medical Sciences Khorramabad Iran; ^6^ Rofeideh Rehabilitation Hospital University of Social Welfare and Rehabilitation Sciences Tehran Iran; ^7^ Nursing Care Research Center, Clinical Sciences Institute Baqiyatallah University of Medical Sciences Tehran Iran

**Keywords:** coronavirus disease 2019, healthcare workers, state anxiety, trait anxiety

## Abstract

**Background and Aims:**

Healthcare workers (HCWs) are in contact with patients afflicted by coronavirus disease 2019 (COVID‐19). The high communicability of COVID‐19 and its different challenges cause varying levels of anxiety for HCWs. The aim of this study was to assess the predictors of state and trait anxiety among HCWs during the COVID‐19 pandemic in Iran.

**Methods:**

This cross‐sectional descriptive‐analytical study was conducted in 2021–2022. Participants were 628 HCWs purposively recruited from educational hospitals in Iran. Data were collected through a demographic and occupational characteristics questionnaire and the State Trait Anxiety Inventory and were analyzed through the SPSS software (v. 23.0).

**Results:**

Most participants were female (70.8%), married (67.4%), and nurses (69.7%), and had a bachelor's degree (64.0%). The means of their age and clinical work experience were 34.69 ± 6.28 years and 9.22 ± 3.98 years, respectively. Most participants suffered from moderate to very severe state (91.7%) and trait (81.2%) anxiety. The mean scores of state and trait anxiety had significant relationship with gender and employment type (*p* < 0.05) and the mean score of state anxiety had significant relationship with marital status and work shift (*p* < 0.05). Employment type and work shift significantly predicted 15% of the total variance of the mean score of state anxiety.

**Conclusion:**

Most HCWs in Iran suffered from moderate to very severe state and trait anxiety during the COVID‐19 pandemic. Strong psychological support is essential for HCWs during epidemics.

**Abbreviations**
COVID‐19: coronavirus disease 2019
HCWs: healthcare workers
SPSS: statistical product and service solutions.
STROBE: strengthening the reporting of observational studies in epidemiology

## 1 Introduction

Coronavirus disease 2019 (COVID‐19) first appeared in December 2019 in Wuhan, China, and rapidly spread throughout the world and turned into a pandemic. The first case of the disease in Iran was formally reported on February 19, 2020 [1].

The rapid spread of COVID‐19 and the high need for the hospitalization of the afflicted patients caused serious challenges for healthcare workers (HCWs) [2, 3]. One of the most important challenges was severe increase in the number of hospitalized patients without proportionate increase in the number of HCWs, which seriously increased HCWs' workload [3, 4]. The extremely heavy workload of HCWs during the COVID‐19 pandemic was, in turn, associated with consequences such as physical fatigue, job burnout, mental health problems, and absence from work [3, 4, 5, 6]. Besides, they experienced problems such as constant fear over affliction by COVID‐19, long work hours, high risk of infection transmission to others, the need for using heavy personal protective equipment, social isolation, separation from family members, and the high risk of affliction by mental health problems, particularly anxiety [3, 4, 7].

Anxiety is the core of different mental disorders and is defined as a possible threat in the future characterized by annoying feelings such as uncertainty, horror, and fear [7, 8]. The physical symptoms of anxiety include palpitation, tachypnea, excessive perspiration, dry mouth, and muscular tension [2, 8]. There are two main types of anxiety, namely state anxiety and trait anxiety [8]. State anxiety is a temporary emotional state which appears due to the immediate conditions and is associated with tension, conflict, and loss of control. Trait anxiety refers to personality‐related anxiety and is a relatively stable characteristic which predisposes individuals to anxiety in most places and situations [8, 9]. Individuals with a higher level of trait anxiety experience more frequent, severe, and longer courses of state anxiety during stressful life events. In other words, trait anxiety is a risk factor for anxiety disorders [10, 11, 12]. Assessment of both state and trait anxiety during pandemics, such as the COVID‐19 pandemic, can provide a better understanding about HCWs' psychological well‐being [7, 8].

Various studies assessed HCWs' experiences and mental health during the COVID‐19 pandemic [2, 3, 4, 5, 6, 7]. A systematic review reported that the prevalence rates of anxiety, depression, and insomnia among HCWs during the COVID‐19 pandemic were respectively 37%, 36%, and 32%, and the prevalence of anxiety and depression were higher among women [13]. Another study found that around 75% of HCWs who provided care to patients with COVID‐19 suffered from moderate to severe anxiety [5]. Moreover, some studies reported that most HCWs attempt to conceal their anxiety in their contacts with patients with COVID‐19 or their families [4, 14, 15].

High levels of anxiety can lead to physical problems (such as tachycardia, hypertension, and musculoskeletal pains), sleep disorders, negative thoughts, a sense of failure and disappointment, altered interpersonal relationships, impaired concentration, and distraction, and reduce the quality of healthcare services [5, 6, 8, 16]. HCWs' anxiety and depression can increase the risk of adverse safety events at work, such as medical errors by 63% [17] and reduce their quality of life [6].

A wide range of factors can affect HCWs' anxiety during the COVID‐19 pandemic. Examples of these factors are age, gender, marital status, fear of infection transmission to others, occupation, and work experience [2, 6, 18, 19]. The effects of these factors on anxiety vary according to HCWs' sociocultural and financial status. Therefore, context‐based studies are necessary to explore HCWs' psychological experiences and the influential factors on their anxiety. Moreover, regular screening is essential to assess their mental health problems, particularly state and trait anxiety [3, 5]. To the best of our knowledge, limited data are available on Iranian HCWs' anxiety during the COVID‐19 pandemic. Therefore, the present study was done to assess the predictors of HCWs' state and trait anxiety during the COVID‐19 pandemic in Iran to help healthcare policy‐makers develop better managerial and protective programs according to the immediate context and culture.

## 2 Methods

### 2.1 Study Design

This cross‐sectional descriptive‐analytical study was conducted from March 2021 to January 2022 based on the Strengthening the Reporting of Observational studies in Epidemiology (STROBE) guideline [20].

### 2.2 Participants and Setting

**Figure 1:**
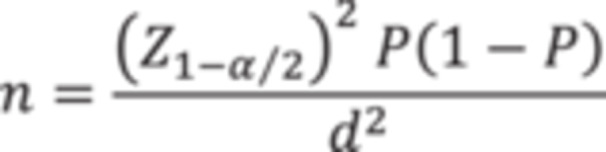
The formula for sample size calculation.

The statistical population of the study comprised HCWs of the hospitals affiliated to Iran University of Medical Sciences, Tehran, Iran. Sample size was calculated with a confidence level of 0.95 and a *d* of 0.035 and using the findings of the Karasu et al. study, which reported that the prevalence of anxiety among HCWs was 75% [5]. The output of the sample size calculation formula (Figure 1) was 600.

Sampling was purposively performed and eligibility criteria were care provision to patients with COVID‐19, a work experience of at least 1 month in COVID‐19 care centers, a clinical work experience of at least 1 year, no history of life crises (such as a significant loss or divorce) during the past 6 months, no self‐report history of using anxiolytic, antidepressant, or psychotropic agents, and agreement for participation. Participants with incomplete answering to the study instruments were excluded.

### 2.3 Instruments

Instruments were a demographic and occupational characteristics questionnaire and the State Trait Anxiety Inventory [21]. The first instrument had nine items on age, gender, marital status, academic degree, occupation, work experience, employment type, work shift, and overtime work hours per month.

The State Trait Anxiety Inventory is a standard instrument with acceptable validity and reliability for anxiety assessment. It has 20 items on state anxiety and 20 items on trait anxiety. Items on state anxiety are scored on a 4‐point Likert scale as follows: 1: “Not at all”; 2: “Somewhat”; 3: “Moderately so”; and 4: “Very much so”. Items on trait anxiety are also scored on a 1–4 Likert scale as follows: 1: “Almost never”; 2: “Sometimes”; 3: “Often”; and 4: “Almost always”. The possible total score of state and trait anxiety is 20–80 and is interpreted as shown in Table 1 [21]. A study in Iran assessed the psychometric properties of the Persian version of the inventory. The study reported that its Cronbach's alpha and test–retest correlation coefficients were 0.846 and 0.889 for state anxiety, and 0.886 and 0.854 for trait anxiety, respectively [22].

**Table 1:** The interpretation of the mean scores of state and trait anxiety.

Anxiety type	Anxiety level	Score range
State anxiety	Mild anxiety	20 < scores < 31
	Moderate anxiety	32 < scores < 53
	Relatively severe anxiety	54 < scores < 64
	Severe anxiety	65 < scores < 75
	Very severe anxiety	Scores > 75
Trait anxiety	Mild anxiety	20 < scores < 31
	Moderate anxiety	32 < scores < 52
	Relatively severe anxiety	53 <scores < 62
	Severe anxiety	63 < scores < 72
	Very severe anxiety	Scores > 72

### 2.4 Data Collection

Participants completed the instruments online and through the self‐report method. The WhatApp, Telegram, and Eitaa applications were used for data collection. Participants read, completed, and signed the informed consent form of the study online.

### 2.5 Data Analysis

Data were analyzed through the Statistical Package for Social Science (SPSS) version 23 software. Frequencies and percentages were used to find the frequency distribution of the study variables. Data normality was tested using the Shapiro‐Wilk's test, which showed the normal distribution of the scores of both state (*p* = 0.126) and trait (*p* = 0.175) anxiety. Data were summarized and presented using the measures of descriptive statistics (namely, frequency distribution, mean, and standard deviation). The relationship of the mean scores of state and trait anxiety with age, work experience, and monthly overtime work hours was examined using the Pearson's correlation analysis. Moreover, the relationship of these mean scores with categorical variables, namely gender, marital status, academic degree, employment type, and work shift, was examined through the independent‐sample *t‐*test and the one‐way analysis of variance. The multiple linear regression analysis with the Stepwise method was used to determine the predictors of state and trait anxiety [23]. The level of significance was set at less than 0.05.

### 2.6 Ethical Considerations

The Ethics Committee of the University of Social Welfare and Rehabilitation Sciences, Tehran, Iran, approved this study (code: IR.USWR.REC.1400.317). Participants completed and signed the online informed consent form of the study, which included information about the authors, aim of the study, confidentiality of the study data, and voluntariness of participation in and withdrawal from the study. They could access and complete the study instruments only if they signed the informed consent form of the study.

## 3 Results

In total, 686 eligible HCWs were recruited to the study. Fifty‐eight participants (8.5%) were excluded because of their incomplete answers to the study instruments, and final data analysis was performed on the data collected from 628 participants. The means of participants' age, clinical work experience, and monthly overtime work hours were 34.69 ± 6.28 years, 9.22 ± 3.98 years, and 52.04 ± 28.06 h, respectively. Most participants were female (70.8%), married (67.4%), and nurses (69.7%), had bachelor's degree (64.0%), and worked rotating shifts (70.3%). Almost half of them secured permanent formal employment (47.7%) (Table 2).

**Table 2:** The relationship of participants' characteristics with the mean scores of their state and trait anxiety.

Characteristics		*N* (%) or mean ± SD	State anxiety mean ± SD	Test result	Trait anxiety mean ± SD	Test result
Gender	Female	445 (70.8)	49.31 ± 11.63	*t* = 0.067	46.76 ± 11.52	*t* = 1.139
	Male	183 (29.2)	47.23 ± 9.83	*p* = 0.02^a^	43.55 ± 9.22	*p* < 0.001^a^
Marital status	Married	423 (67.4)	47.94 ± 11.01	*t* = 0.046	45.00 ± 10.64	*t* = 0.205
	Single	205 (32.6)	46.1 ± 10.03	*p* = 0.03^a^	44.2 ± 9.50	*p* = 0.07^a^
Academic degree	Bachelor's	402 (64.0)	47.22 ± 10.15		44.66 ± 10.23	*F* = 0.893
	Master's and higher	155 (24.6)	46.93 ± 9.55	*F* = 2.131	44.90 ± 9.70	*p* = 0.10^b^
	Doctor of medicine	71 (11.4)	46.67 ± 8.92	*p* = 0.32^b^	44.96 ± 9.41	
Occupation	Physician	71 (11.4)	46.62 ± 9.52	*F* = 0.832	43.00 ± 9.47	*F* = 1.112
	Nurse	438 (69.7)	47.81 ± 9.91	*p* = 0.11^b^	43.63 ± 9.17	*p* = 0.13^b^
	Emergency medicine staff	119 (18.9)	47.09 ± 10.07		43.30 ± 10.91	
Employment type	Permanent official	300 (47.7)	46.9 ± 9.58	*F* = 3.122	43.02 ± 8.02	*F *= 2.992
	Conditional official	95 (15.2)	46.61 ± 9.67		43.18 ± 9.25	
	Contractual	124 (19.7)	46.01 ± 9.28	*p* = 0.01^b^	43.82 ± 8.87	*p* = 0.04^b^
	Mandatory postgraduation service	109 (17.4)	49.82 ± 9.55		45.05 ± 9.87	
Work shift	Day	109 (17.4)	46.75 ± 9.72	*F* = 0.712	43.89 ± 9.90	*F* = 1.682
	Night	77 (12.3)	49.77 ± 9.07	*p* = 0.02^b^	45.23 ± 10.07	*p* = 0.22^b^
	Rotating	442 (70.3)	48.14 ± 9.74		43.04 ± 9.83	
Age (years)		34.69 ± 6.28	47.02 ± 9.38	*r* = 0.04	43.61 ± 9.57	*r* = 0.03
				*p* = 0.52^c^		*p *= 0.43^c^
Work experience (years)		9.22 ± 3.98	47.23 ± 10.09	*r* = 0.01	43.20 ± 9.21	*r* = 0.02
				*p* = 0.62^c^		*p* = 0.58^c^
Overtime work per month (hours)		52.04 ± 28.06	47.08 ± 9.72	*r *= 0.04	43.23 ± 9.51	*r* = 0.02
				*p* = 0.39^c^		*p* = 0.56^c^

^a^ The results of the independent‐sample *t‐*test.

^b^ The results of the one‐way analysis of variance.

^c^ The results of the Pearson's correlation analysis.

The mean scores of participants' state and trait anxiety were 47.23 ± 6.31 and 43.85 ± 7.84, respectively. Most participants reported moderate to very severe state anxiety (91.7%), and around one‐fifth of them reported severe to very severe state anxiety (21.1%). Moreover, most of them reported moderate to very severe trait anxiety (81.2%) and one‐tenth of them reported severe to very severe trait anxiety (10%) (Table 3).

**Table 3:** The severity of participants' state and trait anxiety.

State anxiety		Trait anxiety	
Severity	*N* (%)	Severity	*N* (%)
Mild anxiety	52 (8.3)	Mild anxiety	118 (18.8)
Moderate anxiety	237 (37.7)	Moderate anxiety	246 (39.2)
Relatively severe anxiety	175 (27.9)	Relatively severe anxiety	201 (32.0)
Severe anxiety	117 (18.6)	Severe anxiety	48 (7.6)
Very severe anxiety	47 (7.5)	Very severe anxiety	15 (2.4)
Mean ± SD	47.23 ± 6.31	Mean ± SD	43.85 ± 7.84

The mean scores of state and trait anxiety had no significant relationship with age, academic degree, occupation, clinical work experience, and monthly overtime work hours (*p *> 0.05). However, the mean scores of both state and trait anxiety among female participants were significantly more than their male counterparts (*p *< 0.05). Participants who were performing their mandatory postgraduation service also obtained significantly higher state and trait anxiety scores (*p *< 0.05). Moreover, the mean score of state anxiety among married participants and participants who worked night shift was significantly more than single participants and participants who worked day or rotating shifts, respectively (*p *< 0.05) (Table 2).

The multiple linear regression analysis with the stepwise method showed that employment type and work shift significantly predicted 15% of the total variance of the mean score of state anxiety, and employment type significantly predicted 5% of the total variance of the mean score of trait anxiety (*p *< 0.05) (Table 4).

**Table 4:** The results of the multiple linear regression analysis to determine the predictors of state and trait anxiety.

Anxiety	Predictors	*R*	*R* ^2^	Adjusted *R* ^2^	*B*	SE	Beta	*t*	*p* value
State	Constant	0.391	0.126	0.156	1.926	2.788	—	0.707	0.50
	Employment type				0.188	0.064	0.181	2.771	0.01
	Work shift				0.024	0.009	0.144	2.191	0.04
Trait	Constant	0.272	0.069	0.051	8.425	2.377	—	3.478	0.001
	Employment type				0.114	0.053	0.132	2.074	0.04

## 4 Discussion

This study assessed the predictors of HCWs' state and trait anxiety during the COVID‐19 pandemic in Iran. Findings revealed that the mean score of participants' state anxiety was 47.23 ± 6.31, 91.7% of them suffered from moderate to very severe state anxiety, and 21.1% of them suffered from severe to very severe state anxiety. A study in Indonesia found that the mean score of HCWs' state anxiety was 39.6 ± 11.5% and 56.3% of them suffered from moderate to severe state anxiety [24]. These values in a study on HCWs in Turkey were 51.5 ± 9.9% and 65.5%, respectively [25]. A systematic review and meta‐analysis on 93 studies conducted during the COVID‐19 pandemic on 93112 nurses also found that the total prevalence of anxiety was 37% [26].

State anxiety is a temporary and short‐term emotional state which is associated with mental arousal and apprehension in a specific time and place [7, 8, 10]. The high prevalence of HCWs' state anxiety during the COVID‐19 pandemic may be due to the necessity of long‐term quarantine, fear over affliction by the disease, financial strains, disappointment, limited organizational support, and shortage of medications, equipment, and resources. Although the overall prevalence of HCWs' anxiety increases during infectious disease epidemics, the prevalence and severity of state anxiety in the present study were higher than in previous studies [24, 25, 26]. This may be due to differences among studies concerning their contexts. HCWs in Iran usually face different sociocultural and financial challenges and faced unique unexpected challenges during the COVID‐19 pandemic, including shortage of experienced staff, inappropriate care planning, inadequate organizational support, lack of specialized education about COVID‐19 care, poor interdisciplinary collaboration, delays in governmental decisions for COVID‐19 management, coincidence of COVID‐19 with a high prevalence of H1N1 influenza, and limited public trust in media [1, 3]. Severe international sanctions against Iran also turned COVID‐19 care very difficult so that it was like “swimming with tied hands” [1]. Shortage of medical equipment and limited access to diagnostic kits and COVID‐19 vaccines significantly increased COVID‐19 affliction and mortality rates in Iran and caused high levels of mental disorders such as anxiety among Iranian HCWs [1, 3]. Severe anxiety among HCWs needs effective assessment and management because it negatively affects their health and care quality. Therefore, strategies such as anxiety management education, psychological counseling, and support are essential to reduce their anxiety.

The mean score of trait anxiety in the present study was 43.85 ± 7.84, 81.2% of participants had moderate to very severe trait anxiety, and 10% of them had severe to very severe trait anxiety. In agreement with our findings, a study found that the mean score of HCWs' trait anxiety was 39.4 ± 7.9% and 26.9% of them suffered from high levels of trait anxiety [24]. These values in another study were 44.2 ± 7.7% and 74.3%, respectively [5]. Trait anxiety is a complex phenomenon affected by different personal factors (such as previous negative experiences, childhood traumas, and genetics) and contextual factors (such as society, culture, politics, and economy). High levels of trait anxiety increase the risk of anxiety disorders over time [8, 10, 14] and undermine the quality of life [6]. Therefore, the high level of trait anxiety in the present study is a warning sign for healthcare policy‐makers and highlights the importance of serious interventions to improve HCWs' mental health.

Our findings indicated that state and trait anxiety had no significant relationship with age, educational level, work experience, and monthly overtime work hours. This agrees with the findings of some previous studies which reported that HCWs' anxiety had no significant relationship with age [18, 19], occupation [6, 19], educational level [15], work experience [19], overtime work hours [18], and history of affliction by chronic diseases [2]. However, some studies reported that older and more experienced HCWs had higher levels of anxiety [2], while some studies reported higher levels of anxiety among younger and less experienced HCWs [27, 28]. These contradictions among studies are attributable to the differences among them respecting their settings and participants' sociocultural and financial characteristics, and highlight the necessity of conducting more context‐based studies to determine the predictors of anxiety and develop context‐based interventions to prevent anxiety.

The mean scores of female participants' state and trait anxiety in the present study were significantly more than male participants. Several previous studies also reported the same finding [5, 6, 14]. A study also reported femininity as a significant predictor of anxiety among HCWs [14]. During epidemics, women mostly consider diseases more lethal and more communicable and hence, experience higher levels of anxiety. As women in Iran are the main caregivers of their family members, they may experience higher levels of anxiety over affliction by COVID‐19 and hence, interventions are necessary to improve their mental health.

The mean scores of state and trait anxiety in the present study had a significant relationship with employment type, so that HCWs who were performing their postgraduation mandatory service had higher levels of state and trait anxiety. Similarly, a previous study reported the higher levels of depression, anxiety, and stress among these HCWs [3]. This may be due to the greater workload, lower job security, and unstable occupational status of these HCWs. Studies show that HCWs with unstable occupational status experience higher levels of mental strain due to their lower perceived organizational support and heavier workload [3, 15].

We also found that the mean score of married participants' state anxiety was significantly more than their single counterparts which agrees with the findings of several previous studies [16, 26, 29]. Married HCWs may experience higher levels of anxiety due to their fear over transmitting disease to their spouses and children, their inability to freely visit their families during the quarantine period, and their concern over the future. Moreover, they may have more familial or social needs due to their parental or spousal roles and hence, experience more anxiety in stressful conditions. Meanwhile, single HCWs seem to have less familial responsibility and commitment and hence, may experience lower state anxiety.

Another finding of the present study was the higher mean score of state anxiety among HCWs with night shift. Some previous studies also reported the higher levels of mental disorders among night workers [3, 30]. This may be due to their greater fatigue, inability to have quality sleep, and severe staff shortage on night shifts. Long work hours, inappropriate work schedule, and separation from family are influential factors on HCWs' mental health status [3, 4]. Therefore, improvement of work schedule and reduction of work hours are essential to improve the mental health of HCWs, particularly night workers.

## 5 Strengths and Limitations

This study was conducted at national level and while HCWs in Iran experienced high levels of psychological strain due to the shortage of medications, personal protective equipment, and vaccines approved by the World Health Organization resulting from extensive international sanctions against Iran.

Data collection was performed online due to COVID‐19‐related restrictions. Moreover, the data were collected through the self‐report method and hence, participants' mental status at the time of completing the instruments might have affected their responses. Although the State Trait Anxiety Inventory is a standard instrument for anxiety assessment, the data obtained through it may not be appropriate for diagnosing mental health problems.

## 6 Conclusion

Most HCWs suffer from moderate to very severe state and trait anxiety, and their anxiety correlates with their gender, employment type, marital status, and work shift. Comprehensive interventions for the early diagnosis and management of the symptoms of state and trait anxiety among HCWs can prevent the negative effects of anxiety on them and improve care quality and patient satisfaction. Healthcare managers and policy‐makers are recommended to take the necessary measures to identify the HCWs who are at risk for mental health problems and provide them with psychological counseling services to improve their mental health. Moreover, the findings of the present study can be used to develop programs to improve HCWs' employment type and work schedule.

## Author Contributions

**Azam Sharifi:** conceptualization, data curation, formal analysis, investigation, methodology, writing – original draft, writing – review and editing. **Masoud Fallahi‐Khoshknab:** conceptualization, investigation, methodology, project administration, supervision, writing – review and editing. **Shamaneh Mohammadi:** conceptualization, methodology, writing – review and editing. **Mashaallah Zeraati Nasrabadi:** data curation, writing – review and editing. **Zahra Jamshidi:** data curation, writing – review and editing. **Mohsen Aghabeygi‐ Arani:** data curation, writing – review and editing. **Nilofar Mirzaei:** data curation, writing – review and editing. **Negin Fallahi‐Khoshknab:** data curation, writing – review and editing. **Parisa Rasouli:** data curation, writing – review and editing. **Abbas Ebadi:** formal analysis, methodology, writing – review and editing.

## Ethics Statement

The Ethics Committee of the University of Social Welfare and Rehabilitation Sciences, Tehran, Iran, approved this study with the code of IR.USWR. REC.1400.317.

## Conflicts of Interest

The authors declare no conflicts of interest.

## Transparency Statement

The corresponding author, Masoud Fallahi‐Khoshknab, affirms that this manuscript is an honest, accurate, and transparent account of the study being reported; that no important aspects of the study have been omitted; and that any discrepancies from the study as planned (and, if relevant, registered) have been explained.

## Data Availability

The data that support the findings of this study are available on request from the corresponding author. The data is not publicly available due to privacy or ethical restrictions.
